# Tcf-3 expression and **β** -catenin mediated transcriptional activation in aggressive fibromatosis (desmoid tumour)

**DOI:** 10.1054/bjoc.2001.1857

**Published:** 2001-07

**Authors:** S Tejpar, C Li, C Yu, R Poon, H Denys, R Sciot, E Van Cutsem, J J Cassiman, B A Alman

**Affiliations:** The Program in Developmental Biology, The Hospital for Sick Children and the University of Toronto, 555 University Avenue, Toronto, Ontario M5G1X8, Canada; The Center for Human Genetics; Department of Pathology; Department of Gastroenterology, Katholieke Universiteit Leuven, Herestraat 49, Leuven, 3000, Belgium; The Department of Surgery and Division of Orthopaedic Surgery, The Hospital for Sick Children and the University of Toronto, 555 University Avenue, Toronto, Ontario M5G1X8, Canada

**Keywords:** β-catenin, tcf/lef transcription factors, aggressive fibromatosis, APC

## Abstract

Aggressive fibromatosis harbours mutations resulting in β-catenin protein stabilization. Primary cell cultures demonstrate constitutive tcf activation in aggressive fibromatosis. Expression and co-immunoprecipitation studies suggest that β-catenin binds and activates tcf-3 in this tumour. This is the first demonstration of tcf-3 activation by β-catenin stabilization in a human neoplastic process. © 2001 Cancer Research Campaign http://www.bjcancer.com


British Journal of Cancer (2001) 85(1), 98–101
© 2001 Cancer Research Campaign
doi: 10.1054/ bjoc.2001.1857, available online at http://www.idealibrary.com on IDEAL®

http://www.bjcancer.com

### Tcf-3 expression and β-catenin mediated transcriptional activation in aggressive fibromatosis (desmoid tumour)

**S Tejpar[1,2], C Li[1], C Yu[1], R Poon[1], H Denys[2], R Sciot[3], E Van Cutsem[4], JJ Cassiman[2] and BA Alman[1,5]**

[1]The Program in Developmental Biology, The Hospital for Sick Children and the University of Toronto, 555 University Avenue, Toronto, Ontario, M5G1X8, Canada; [2]The Center for Human Genetics, [3]Department of Pathology, and [4]Department of Gastroenterology, Katholieke Universiteit Leuven, Herestraat 49, 3000, Leuven, Belgium; [5]The Department of Surgery and Division of Orthopaedic Surgery, The Hospital for Sick Children and the University of Toronto, 555 University Avenue, Toronto, Ontario, M5G1X8, Canada

**Summary** Aggressive fibromatosis harbours mutations resulting in β-catenin protein stabilization. Primary cell cultures demonstrate constitutive tcf activation in aggressive fibromatosis. Expression and co-immunoprecipitation studies suggest that β-catenin binds and activates tcf-3 in this tumour. This is the first demonstration of tcf-3 activation by β-catenin stabilization in a human neoplastic process. © 2001 Cancer Research Campaign http://www.bjcancer.com

**Keywords**: β-catenin; tcf/lef transcription factors; aggressive fibromatosis; APC

Aggressive fibromatosis is a locally invasive soft tissue lesion composed of a clonal proliferation of spindle (fibroblast-like) cells (Alman et al, 1997a). It harbours somatic mutations in either the adenomatous polyposis coli (APC) gene or the β-catenin gene, resulting in β-catenin protein stabilization (Alman et al, 1997b; Li et al, 1998; Tejpar et al, 1999). β-catenin is located in the nucleus in aggressive fibromatosis, suggesting a role in nuclear transcription (Tejpar et al, 1999).

β-catenin is a key signalling molecule in the Wnt signalling pathway. Activation of the pathway results in β-catenin protein stabilization. When stabilized, it binds to members of the t-cell factor-lymphoid enhancer factor (tcf-lef) family, forming a transcriptional activating complex. Mutations in β-catenin, removing amino terminal phosphorylation sites, or mutations in the members of a multi-protein complex that aids in β-catenin degradation, such as APC, also cause β-catenin stabilization (Miller et al, 1999).

Tcf/lef family members are architectural transcription factors, whose ability to transactivate transcription is altered by binding to β-catenin. Each of the tcf/lef transcription factors has a slightly different structure, and is expressed at different times and by different cells during normal development. Despite the difference in structure, all bind and activate the same consensus DNA sequence, suggesting that the structural differences allow for specific binding of other proteins with a repressor or enhancer function (Roose and Clevers, 1999a; Barker et al, 2000). This could result in the activation of different target genes. Murine data suggest that this is true for the skin, in which tcf-3 and lef-1 do not transactivate transcription in the same manner, thought due to the presence of specific proteins that bind tcf-3 altering its activation (Gat et al, 1998).

*Received 18 January 2001*
*Revised 22 March 2001*
*Accepted 27 March 2001*

*Correspondence to:* BA Alman

Although β-catenin is stabilized in a variety of neoplastic processes, such as colonic neoplasia, the effects of stabilization vary between tumour types, as colonic polyps can go on to malignancy, while aggressive fibromatosis only very rarely progresses to a malignant process. One possibility for the difference in behaviour is that stabilized β-catenin binds different transcription factors, which will activate transcription in a different manner. Binding to different transcription factors is already demonstrated between colonic neoplasia (in which β-catenin binds tcf-4) (Morin et al, 1997) and pilomatricomas (in which β-catenin binds lef-1) (Chan et al, 1999).

### METHODS

### Patients and case material

Tumours and normal tissues from 10 patients with sporadic aggressive fibromatoses were studied. 6 tumours had β-catenin mutations (substitution of threonine at codon 41), 2 had APC mutations (one had a frameshift at codon 1567 and the other a frameshift at codon 1371, both resulting in an early stop codon), and 2 had no mutations identified in either β-catenin or APC. Normal fascial tissue from the resection margins were used as controls. RNA was extracted from 5 colon cancers, and analysed for transcription factor expression in an identical manner to the fibromatoses. Control cell lines, were the SW480 (colorectal cancer cell line, APC mutant producing a high level of β-catenin protein), HCT116 (colorectal cancer cell line, β-catenin mutant), CRL1790 (normal colonic epithelium), Jurkat (T-cell lymphoma) and Molt-4 (acute lymphoblastic leukaemia).

### Tcf transcriptional activation

Primary cell cultures from the aggressive fibromatosis cases and normal fibrous tissues were transiently transfected after their first passage in culture. The SW480 colon cancer cell line was used as a control. Cells in 35 mm dishes were transiently transfected in triplicate with 1000 ng of the luciferase reporter construct pTOPFLASH or pFOPFLASH. In addition some cell dishes were also co-transfected with either 1000 ng ΔN-Tcf-4E; 1000 ng of ΔN89 β-catenin (Munemitsu et al, 1996); 1000 ng of the full length APC gene; or empty vector controls. 3 $\mu$l of FuGENE Transfection Reagent (Boehringer, Mannheim) was used according to the manufacturer's protocol, and in all cases, 500 ng of Rous Sarcoma Virus β-galactosidase DNA was also transfected as a control for transfection efficiency. Cells were harvested 24–36 hours after the transfection, and luciferase reporter enzyme activity was measured and normalized to β-2 galactosidase. The pTOPFLASH reporter construct contains copies of the consensus binding sequence for tcf transcription factors linked to a luciferase reporter and the pFOPFLASH reporter construct contains a mutated binding sequence linked to the reporter as a control (Morin et al, 1997). In the case of the SW480 cell line and the aggressive fibromatoses containing APC mutations, the wild-type APC gene in a CMV vector was transfected. In all of the tumours a dominant negative tcf, ΔN tcf- 4E (Tetsu and McCormick, 1999), was also transfected. ΔN tcf- 4E binds the tcf consensus sequence, but prevents transcriptional activation by other transcription factors. A ratio of the normalized pTOPFLASH/pFOPFLASH luminescence was calculated for each cell dish. Means and standard deviations were determined for each cell type and transfection condition, and compared using the 2 way *t*-test.

### Transcription factor expression

Total RNA was isolated using TRIzol reagents (Gibco), and analysed using RT-PCR and Northern analysis. RT-PCR was performed using previously described primers and conditions for expression of Tcf-1, Lef-1, Tcf-3, and Tcf-4 (Zhou et al, 1995; Korinek et al, 1997). Northern analysis was performed using 32P-labelled PCR generated probes. GAPDH expression was measured as a loading control.

### Co-immunoprecipitation with tcf antibodies

Monoclonal antibodies that recognize either tcf-1, tcf-4, or one that recognizes both tcf-3 and tcf-4 (tcf-3/4 antibody) were utilized for co-immunoprecipitation (Barker et al, 1999). Total protein lysates were isolated using native conditions from an aggressive fibromatosis primary cell cultures and from the SW480 cell line. The lysates were then incubated with additional buffer buffer (1% Triton X-100, 150 mM NaCl, 10 mM Tris pH 7.4, 1 mM EDTA, 1 mM EGTA pH 8.0, 0.2 mM sodium ortho-vanadate, 0.2 mM PMSF, 0.5% NP-40), and one of the monoclonal antibodies for one hour at 4˚C. The antibodies were recovered using Protein A plus agarose. The immunoprecipitates were subjected to Western analysis for β-catenin as previously reported (Tejpar et al, 1999).

### RESULTS

### Constitutive β-catenin mediated tcf-dependent transcriptional activation in aggressive fibromatosis

A significant increase in the pTOPFLASH activity was observed in aggressive fibromatosis cultures over that in primary fibroblast cell cultures from the same patients (ratio of 12.5 vs 2.5, $P < 0.05$, shown in Figure 1). Our previous work showed that APC transfection into aggressive fibromatosis primary cultures that contained an APC mutation reduced β-catenin protein level (Li et al, 1998). Transfection of wild-type APC these cultures also resulted in a significant decline in pTOPFLASH activity. When a stabilized form of β-catenin, Δ-N-89 β-catenin that lacks the amino terminus phosphorylation sites necessary for protein degradation (Munemitsu et al, 1996), was transfected into either normal fibroblasts, or the aggressive fibromatoses, it resulted in a doubling of pTOPFLASH/pFOPFLASH ratios (3.1 to 6.8 for the normal fibroblasts and 12.5 to 20.5 for aggressive fibromatosis cell cultures). These data illustrate that β-catenin stabilization is responsible for regulating the tcf-mediated transcriptional activation in this cell type. The dominant negative tcf, ΔN-tcf-4E, (He et al, 1999; Tetsu and McCormick, 1999), was co-transfected into these cultures, resulting in a significant decline of transcriptional activation to a level about the same as for normal fibroblasts (Figure 1).

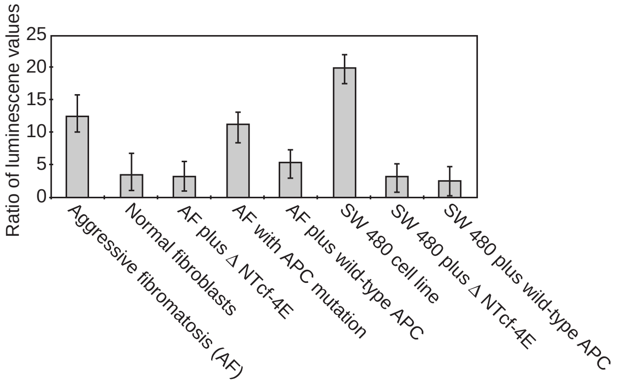

**Figure 1** Tcf transcriptional activation in primary cell cultures from aggressive fibromatosis and normal fibrous tissue cells. Ratio of luminescence is the ratio of pTOPFLASH/pFOPFLASH when normalized for transfection efficiency. Each bar gives the mean and standard deviation for the specific cell type tested. For instance the aggressive fibromatosis bar gives the mean and standard deviation for all of the aggressive fibromatosis samples, while the AF with APC mutation gives the mean and standard deviations for the cases of aggressive fibromatosis with an APC mutation. A higher ratio indicates a higher level of tcf transcriptional activation, with cells exhibiting no transcriptional activation having a ratio of one. Aggressive fibromatosis exhibits a high level of tcf transcriptional activation, close to that of the SW480 colon cancer cell line. Normal fibroblast cultures from the same patients exhibit a significantly lower level of activation ($P < 0.05$), and transfection of the dominant negative tcf into any of the cultures, or transfection of the full length APC gene into a tumour culture with an APC mutation, resulted in a level of activation similar to that detected in the normal fibroblast cultures

### Aggressive fibromatosis expresses tcf-3

Tcf-3 was expressed in all 10 aggressive fibromatoses, as shown in Figure 2. Tcf-4 was expressed in only 3 of the 10 aggressive fibromatoses, while tcf-1 and lef-1 were not expressed. This is in contrast to colon cancers, which express tcf-4 and tcf-1. Normal fibrous tissues from the 10 aggressive fibromatosis patients (fascia from the resection margin) all expressed tcf-3 using RT-PCR, but did not express any of the other transcription factors (data not shown).

### β-catenin binds tcf-3 in aggressive fibromatosis

Western analysis shows β-catenin protein presence in the immunoprecipite using a tcf-3/4 antibody, but its absence in the immunoprecipitate using a tcf-4 antibody (Figure 3). This is in contrast to results from the SW480 colon cancer cell line extracts, in which tcf-4 immunoprecipitates contained β-catenin protein (Figure 3).

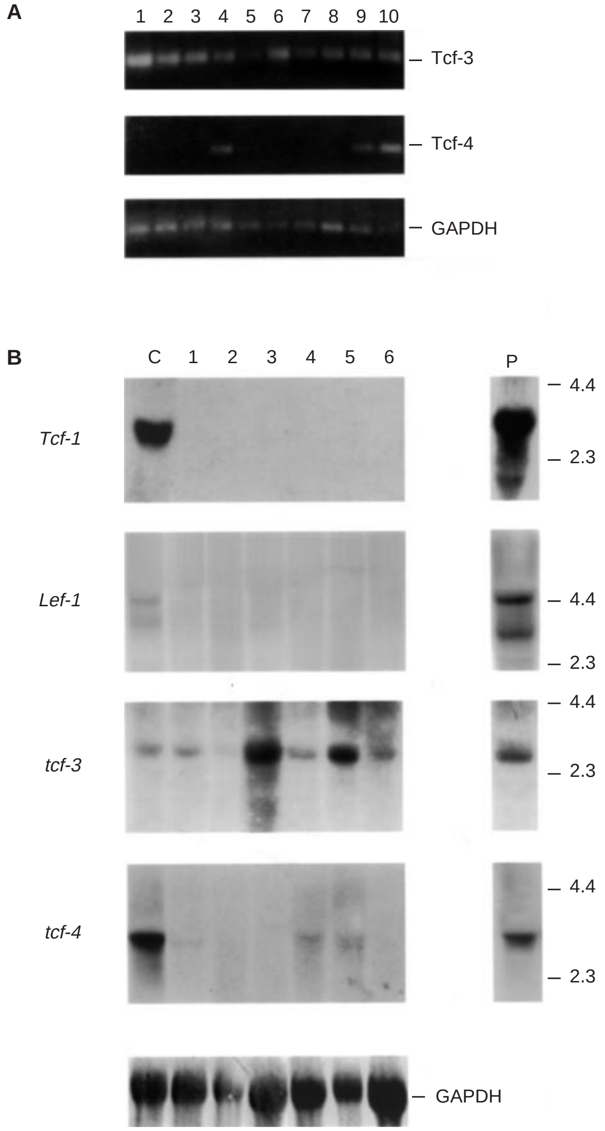

**Figure 2** Tcf/lef expression in aggressive fibromatosis. (**A**) RT-PCR for tcf-3 and tcf-4 from all 10 tumours shows that only 3 express tcf-4. GAPDH expression is used as a control. (**B**) Northern analysis for lef-1, tcf-1, 3 and 4, and GAPDH (as a loading control) from a single membrane containing RNA loaded from one colonic cancers (lane C) and 5 aggressive fibromatoses (lanes 1 to 5). Lane P is loaded with RNA from an appropriate cell line for a positive control. The Jurkat line was used for tcf-1, the Molt-4 for LEF and tcf-3, and the HCT-116 for tcf-4. The only transcription factor that is expressed by all of the fibromatoses is tcf-3

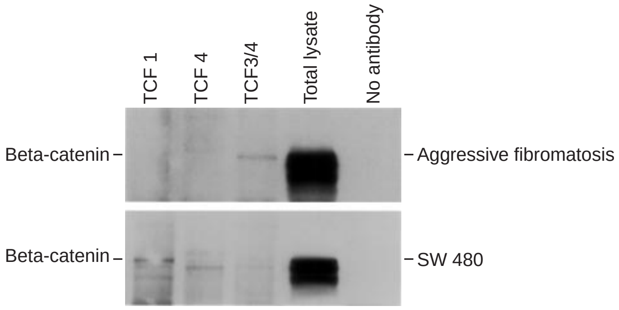

**Figure 3** β-catenin binds tcf-3. Western analysis for β-catenin from tcf antibody co-immunoprecipitates. Lanes are loaded with immunoprecipitates using the various antibodies as labelled, with total protein lysate (positive control), or with an immunoprecipitate obtained without the use of an antibody (negative control). β-catenin is detected in the tcf3/4 immunoprecipitate in aggressive fibromatosis, but not in the tcf-4 immunoprecipitate, suggesting β-catenin binding to tcf-3 in this tumour. This is in contrast to the immunoprecipitates from the SW 480 cell line, in which β-catenin is detected in the immunoprecipitate obtained using the tcf-4 antibody.

### DISCUSSION

We directly demonstrate constitutive transcriptional activation of the tcf/lef family of transcription factors in aggressive fibromatosis. Furthermore, we show that this activation is related to β-catenin stabilization. In combination with our previous data (Alman et al, 1997b; Li et al, 1998; Tejpar et al, 1999), this gives strong evidence that β-catenin mediated tcf-dependent transcription plays a key role in the pathogenesis of aggressive fibromatosis.

Tcf-3 was expressed in all of the cases of aggressive fibromatosis and in normal fibrous tissue from these same patients. This is in contrast to colonic neoplasia which our data, as well as data from other researchers (Korinek et al, 1997; Morin et al, 1997), show express tcf-4; and pilomatricomas, in which lef-1 is expressed (Chan et al, 1999). Each of the tcf/lef family may regulate transcription in a slightly different way (Behrens et al, 1996; Morin et al, 1997). Xenopus studies suggest that tcf-3 can be bound selectively by specific repressors such as C-terminal binding protein (Brannon et al, 1999). Interactions unique to tcf-3 could be responsible for the expression of different β-catenin mediated target genes in aggressive fibromatosis, partially explaining the difference in cell behaviour observed between aggressive fibromatosis and other neoplastic processes exhibiting β-catenin stabilization.

We found that tcf-dependent transcriptional activation in aggressive fibromatosis and normal fibocytes is significantly upregulated by β-catenin stabilization. This is demonstrated by the repression of transcriptional activation with transfection of the full length *APC* in *APC* mutant tumours, which leads to a drop in β-catenin levels; and by the demonstration that overexpression of a stabilized form of β-catenin enhances activation. Normal fibroblasts derived from the same patients with aggressive fibromatosis do not contain a mutation resulting in β-catenin stabilization, but do express tcf-3. These fibroblasts do not exhibit tcf-dependent transcriptional activation, suggesting that β-catenin stabilization is essential for tcf-3 transcriptional activation.

Tcf-1 is a negative regulator of β-catenin mediated tcf-dependent transcriptional activation in colonic epithelial cells. In these cells it is upregulated by tcf-4 mediated transcription (Roose et al, 1999). The lack of tcf-1 in aggressive fibromatosis suggests that this negative regulatory feedback may be lost in aggressive fibromatosis, contributing to β-catenin mediated transcriptional activation.

Some of the tcf/lef target genes identified in colon cancer are not expressed in aggressive fibromatosis. In this study, we found that the target gene, tcf-1 (Roose et al, 1999) was not expressed in aggressive fibromatosis. In another study (Poon, et al 2000), we found that another β-catenin-tcf target gene identified in colonic neoplasia, PPAR-δ (He et al, 1999), was also not upregulated in aggressive fibromatosis. While there are several potential explanations for the difference in expression of these target genes, one possibility is that the different profile of tcf/lef family member expression is responsible for the difference in expression. This difference in expression of target genes may be one factor responsible for the difference in tumour behaviour between different

 © 2001 Cancer Research Campaign neoplastic processes exhibiting β-catenin mediated tcf-dependent transcriptional activation.

### ACKNOWLEDGEMENTS

We thank H Clevers, K Kinzler, F McCormick, P Polakis, O Tetsu, and B Vogelstein for plasmids and reagents. This work was funded by Grants from the National Cancer Institute of Canada with Funds from the Terry Fox Run (9129) and from the Medical Research Council of Canada (MT15136) to BA Alman. BA Alman holds a Canadian Research Chair. S Tejpar was funded by the Fund for Scientific Research-Flanders (FSR).

### REFERENCES

Alman BA, Pajerski ME, Diaz-Cano S, Corboy K and Wolfe HJ (1997a) Aggressive fibromatosis (desmoid tumor) is a monoclonal disorder. *Diagn Mol Pathol* **6**: 98–101

Alman BA, Li C, Pajerski ME, Diaz-Cano S and Wolfe HJ (1997b) Increased β-catenin protein and somatic APC mutations in sporadic aggressive fibromatosis (desmoid tumors). *Am J Pathol* **151**: 329–334

Barker N, Huls G, Korinek V and Clevers H (1999) Restricted high level expression of TCF-4 protein in intestinal and mammary gland epithelium. *Am J Pathol* **154**: 29–35

Barker N, Morin PJ and Clevers H (2000) The Yin-Yang of TCF/β-catenin signaling. *Adv Cancer Res* **77**: 1–24

Behrens J, von Kries JP, Kuhl M, Bruhn L, Wedlich D, Grosschedl R and Birchmeier W (1996) Functional interaction of β-catenin with the transcription factor LEF-1. *Nature* **382**: 638–642

Brannon M, Brown JD, Bates R, Kimelman D and Moon (1999) XCtBP is a XTcf-3 co-repressor with roles throughout Xenopus development. *Development* **126**: 3159–3170

Chan EF, Gat U, McNiff JM and Fuchs E (1999) A common human skin tumor is caused by activating mutations in β-catenin. *Nat Genet* **21**: 410–413

He TC, Chan TA, Vogelstein B and Kinzler KW (1999) PPAR-δ is an APC-regulated target of nonsteroidal anti-inflammatory drugs. *Cell* **99**: 335–345

Korinek V, Barker N, Morin PJ and van Wichen D (1997) Constitutive transcriptional activation by a β-catenin-Tcf complex in APC−/−colon carcinoma. *Science* **275**: 1784–1787

Li C, Bapat B and Alman BA (1998) Adenomatous polyposis coli gene mutation alters proliferation through its β-catenin-regulatory function in aggressive fibromatosis (desmoid tumor). *Am J Pathol* **153**: 709–714

Miller JR, Hocking AM, Brown JD and Moon RT (1999) Mechanism and function of signal transduction by the Wnt/β-catenin and Wnt/Ca2+ pathways. *Oncogene* **18**: 7860–7872

Morin PJ, Sparks AB, Korinek V, Barker N, Clevers H, Vogelstein B and Kinzler KW (1997) Activation of β-catenin-TCF signaling in colon cancer by mutations in β-catenin or APC. *Science* **275**: 1787–1790

Munemitsu S, Albert I, Rubinfeld B and Polakis P (1996) Deletion of an amino-terminal sequence beta-catenin in vivo and promotes hyperphosporylation of the adenomatous polyposis coli tumor suppressor protein. *Mol Cell Biol* **16**: 4088–4094

Poon R, Smits R, Li C, Jagmohan-Changur S, Kong M, Cheon S, Yu C, Fodde R and Alman B (2001) Cyclooxygenase-two (COX-2) modulates proliferation in aggressive fibromatosis (desmoid tumor). *Oncogene* **20**: 451–460

Roose J and Clevers H (1999) TCF transcription factors: molecular switches in carcinogenesis. *Biochim Biophys Acta* 1424(2–3): M23–M37

Roose J, Huls G, van Beest M, Moerer P, van der Horn K, Goldschmeding R, Logtenberg T and Clevers H (1999) Synergy between tumor suppressor APC and the β-catenin-Tcf4 target Tcf1. *Science* **285**: 1923–1926

Sagara N and Katoh M (2000) Mitomycin C resistance induced by TCF-3 overexpression in gastric cancer cell line MKN28 is associated with DT-diaphorase down-regulation. *Cancer Res* **60**: 5959–5962

Tejpar S, Nollet F, Li C, Wunder JS, Michils G, dal Cin P, Van Cutsem E, Bapat B, van Roy F, Cassiman JJ and Alman BA (1999) Predominance of β-catenin mutations and β-catenin dysregulation in sporadic aggressive fibromatosis (desmoid tumor). *Oncogene* **18**: 6615–6620

Tetsu O and McCormick F (1999) β-catenin regulates expression of cyclin D1 in colon carcinoma cells. *Nature* **398**: 422–426

Zhou P, Byrne C, Jacobs J and Fuchs E (1995) Lymphoid enhancer factor 1 directs hair follicle patterning and epithelial cell fate. *Genes Dev* **9**: 700–713

© 2001 Cancer Research Campaign 

## References

[PDF_00362] Alman B. A., Li C., Pajerski M. E., Diaz-Cano S., Wolfe H. J. (1997). Increased beta-catenin protein and somatic APC mutations in sporadic aggressive fibromatoses (desmoid tumors).. Am J Pathol.

[PDF_00359] Alman B. A., Pajerski M. E., Diaz-Cano S., Corboy K., Wolfe H. J. (1997). Aggressive fibromatosis (desmoid tumor) is a monoclonal disorder.. Diagn Mol Pathol.

[PDF_00365] Barker N., Huls G., Korinek V., Clevers H. (1999). Restricted high level expression of Tcf-4 protein in intestinal and mammary gland epithelium.. Am J Pathol.

[PDF_00368] Barker N., Morin P. J., Clevers H. (2000). The Yin-Yang of TCF/beta-catenin signaling.. Adv Cancer Res.

[PDF_00370] Behrens J., von Kries J. P., Kühl M., Bruhn L., Wedlich D., Grosschedl R., Birchmeier W. (1996). Functional interaction of beta-catenin with the transcription factor LEF-1.. Nature.

[PDF_00373] Brannon M., Brown J. D., Bates R., Kimelman D., Moon R. T. (1999). XCtBP is a XTcf-3 co-repressor with roles throughout Xenopus development.. Development.

[PDF_00376] Chan E. F., Gat U., McNiff J. M., Fuchs E. (1999). A common human skin tumour is caused by activating mutations in beta-catenin.. Nat Genet.

[PDF_00378] He T. C., Chan T. A., Vogelstein B., Kinzler K. W. (1999). PPARdelta is an APC-regulated target of nonsteroidal anti-inflammatory drugs.. Cell.

[PDF_00380] Korinek V., Barker N., Morin P. J., van Wichen D., de Weger R., Kinzler K. W., Vogelstein B., Clevers H. (1997). Constitutive transcriptional activation by a beta-catenin-Tcf complex in APC-/- colon carcinoma.. Science.

[PDF_00383] Li C., Bapat B., Alman B. A. (1998). Adenomatous polyposis coli gene mutation alters proliferation through its beta-catenin-regulatory function in aggressive fibromatosis (desmoid tumor).. Am J Pathol.

[PDF_00386] Miller J. R., Hocking A. M., Brown J. D., Moon R. T. (1999). Mechanism and function of signal transduction by the Wnt/beta-catenin and Wnt/Ca2+ pathways.. Oncogene.

[PDF_00389] Morin P. J., Sparks A. B., Korinek V., Barker N., Clevers H., Vogelstein B., Kinzler K. W. (1997). Activation of beta-catenin-Tcf signaling in colon cancer by mutations in beta-catenin or APC.. Science.

[PDF_00392] Munemitsu S., Albert I., Rubinfeld B., Polakis P. (1996). Deletion of an amino-terminal sequence beta-catenin in vivo and promotes hyperphosporylation of the adenomatous polyposis coli tumor suppressor protein.. Mol Cell Biol.

[PDF_00396] Poon R., Smits R., Li C., Jagmohan-Changur S., Kong M., Cheon S., Yu C., Fodde R., Alman B. A. (2001). Cyclooxygenase-two (COX-2) modulates proliferation in aggressive fibromatosis (desmoid tumor).. Oncogene.

[PDF_00399] Roose J., Clevers H. (1999). TCF transcription factors: molecular switches in carcinogenesis.. Biochim Biophys Acta.

[PDF_00401] Roose J., Huls G., van Beest M., Moerer P., van der Horn K., Goldschmeding R., Logtenberg T., Clevers H. (1999). Synergy between tumor suppressor APC and the beta-catenin-Tcf4 target Tcf1.. Science.

[PDF_00404] Sagara N., Katoh M. (2000). Mitomycin C resistance induced by TCF-3 overexpression in gastric cancer cell line MKN28 is associated with DT-diaphorase down-regulation.. Cancer Res.

[PDF_00408] Tejpar S., Nollet F., Li C., Wunder J. S., Michils G., dal Cin P., Van Cutsem E., Bapat B., van Roy F., Cassiman J. J. (1999). Predominance of beta-catenin mutations and beta-catenin dysregulation in sporadic aggressive fibromatosis (desmoid tumor).. Oncogene.

[PDF_00411] Tetsu O., McCormick F. (1999). Beta-catenin regulates expression of cyclin D1 in colon carcinoma cells.. Nature.

[PDF_00413] Zhou P., Byrne C., Jacobs J., Fuchs E. (1995). Lymphoid enhancer factor 1 directs hair follicle patterning and epithelial cell fate.. Genes Dev.

